# HCV core antigen plays an important role in the fight against HCV as an alternative to HCV‐RNA detection

**DOI:** 10.1002/jcla.23755

**Published:** 2021-03-31

**Authors:** Yuhan Wang, Wang Jie, Jiang Ling, Huang Yuanshuai

**Affiliations:** ^1^ Department of Transfusion The Affiliated Hospital of Southwest Medical University Luzhou China

**Keywords:** anti‐HCV, core antigen, diagnostic performance, HCV, HCV‐RNA

## Abstract

**Objective:**

To discuss the clinical significance of HCV‐cAg testing in the diagnosis, activity determination, and monitoring of therapeutic effectiveness of HCV infection and its advantages compared with HCV‐RNA and anti‐HCV antibodies detection.

**Methods:**

By summarizing the published literature, the advantages and significance of HCV core antigen detection were sought.

**Results:**

The expression of HCV‐cAg is highly consistent with that of HCV‐RNA, but compared with HCV‐RNA, detection of HCV‐cAg is easy to operate, time saving, and low cost. HCV‐cAg can be detected within 12~15 days after infection, and the window period can be shortened by5~7 weeks. HCV‐cAg is a serological indicator of virus replication, which can distinguish previous infection of HCV or current infection. HCV‐cAg detection is more suitable for immunocompromised, hemodialysis, organ transplant patients. HCV‐cAg also can be used to monitor antiviral efficacy and predict sustained virological response (SVR).

**Conclusion:**

HCV core antigen has similar clinical sensitivity to NAT and can be used as a substitute for HCV‐RNA in the diagnosis of virus infection. Combined detection of HCV‐cAg and antibody serology can help doctors detect HCV infection earlier, accurately diagnose different stages of HCV infection, and evaluate the therapeutic effect of antiviral drugs, which are beneficial in the prevention and treatment of hepatitis C.

## Graphical abstract

This article aims to discuss the clinical significance of HCV‐cAg testing in the diagnosis, activity determination, and monitoring of therapeutic effectiveness of HCV infection and its advantages compared with HCV‐RNA and anti‐HCV antibodies detection. Through research and summary analysis of a large number of data revealed that the expression of HCV‐cAg is highly consistent with that of HCV‐RNA, but compared with HCV‐RNA, detection of HCV‐cAg is easy to operate, time saving, and low cost. HCV‐cAg can be detected within 12~15 days after infection, and the window period can be shortened by5~7 weeks. HCV‐cAg is a serological indicator of virus replication, which can distinguish previous infection of HCV or current infection. HCV‐cAg detection is more suitable for immunocompromised, hemodialysis, organ transplant patients. HCV‐cAg also can be used to monitor antiviral efficacy and predict sustained virological response (SVR).

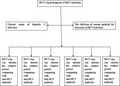

## 1 INTRODUCTION

Hepatitis C is a viral hepatitis caused by infection with the hepatitis C virus (HCV). It is often described as a “silent killer,” mainly because the virus has a long incubation period and the disease is prone to become chronic. Chronic HCV infection can cause varying degrees of damage to the liver. If left untreated, it can lead to a series of complications, such as fibrosis, cirrhosis, and even liver cancer. World Health Organization (WHO) estimated that in 2015, 71 million persons were living with chronic HCV infection worldwide (global prevalence: 1%) and that 399,000 had died from cirrhosis or hepatocellular carcinoma (HCC),^1^ with most of these concentrated in developing countries.^2^ Due to the high missing reporting rate, the incidence of HCV infection is actually higher. HCV infection has become a serious social and public health problem because of its great harm to the health and safety of patients.^3^ The WHO says that unless urgent action is taken to improve access to treatment, chronic hepatitis will cause around 10 million deaths in China by 2030, most of which could be prevented (http://www.wpro.who.int/china/mediacentre/releases/2016/20160727‐china‐world‐hepatitis‐day/en/). In May 2016, the World Health Assembly approved the Global Health Sector Strategy (GHSS) on viral hepatitis, proposing eliminating viral hepatitis as a public health hazard by 2030 (90% reduction in morbidity and 65% reduction in mortality). To eliminate viral hepatitis, 90% of those infected are required to be diagnosed and 80% of those diagnosed are required to receive treatment (global health sector strategy on viral hepatitis 2016–2021. World Health Organization. https://www.who.int/hepatitis/strategy2016‐2021/ghss‐hep/en/). However, in 2015, there were large deficits in achieving these service coverage objectives. Of the 71 million persons with HCV infection, 14 million (20%) had been diagnosed, and of the 14 million diagnosed, 1.1 million (7%) had been started on treatment.^1^

## 2 HCV‐cAg in diagnosis of HCV infection

### 2.1 The deficient of current methods for detection of HCV infection

The symptoms of HCV infection are usually not easily detected. In addition, because HCV is a single positive‐stranded RNA virus, it is easy to mutate and difficult to get a vaccine. There were difficulties in vaccination and fewer screening methods than for HBV. At present, commonly used detection methods include serological detection (HCV core antigen detection/anti‐HCV detection) and molecular biological detection (HCV‐RNA detection). Anti‐HCV antibodies detection is currently the most commonly used screening tool for HCV, but it has some shortcomings: The window period of anti‐HCV detection is long, acute hepatitis C patients are easy to be missed; it cannot distinguish between those who have recovered from previous infection and those who are chronically infected; patients with autoimmune disease may have false‐negative anti‐HCV test results; HCV antibodies are lifelong carriers and cannot be used to monitor antiviral efficacy^4^; among populations with low (<10%) prevalence of HCV infection, assays for anti‐HCV antibodies show high false‐positive rates,^5^ which require confirmation with other more specific supplementary tests.

Because chronic HCV infection is mostly asymptomatic until late clinical stage, there is an urgent need to detect active HCV infection through simple and repeatable methods. For this purpose, clinical guidelines recommend looking for HCV ribonucleic acid (HCV‐RNA) after detection of anti‐HCV antibodies. Quantitative polymerase chain reaction (PCR) of HCV‐RNA can be used for confirmation of active HCV infection, baseline viral load analysis before antiviral treatment, and response assessment during and after antiviral treatment. However, RNA is easy to be degraded (due to the presence of a large number of RNA enzymes in the environment, RNA is very easy to be degraded and prone to false‐negative results). Test samples should not be placed at room temperature after collection. They should be stored at a low temperature immediately. Therefore, a cryogenic refrigerator should be prepared, and RNA must be extracted within 2 hours after blood is drawn. In addition, the RNA extraction process is complex and requires high requirements for operators. From what has been discussed above, HCV‐RNA quantification tests are expensive, time‐consuming, and require highly trained personnel.^6^

The sequence of HCV core antigen (cAg) is highly conserved among all the different viral genotypes. The presence of circulating antigen is associated with complete viral particles but may also be derived from the presence of antigen/antibody complexes and a release of “RNA‐free” antigen by hepatocytes that go into processes of immune‐mediated lysis or apoptosis.^7^ Features of HCV‐cAg detection including HCV‐cAg can be detected within 12~15 days after infection, and the window period can be shortened by 5~7 weeks. HCV‐cAg is a serological indicator of virus replication, which can distinguish previous infection of HCV or current infection. Compared with anti‐HCV antibody test, HCV‐cAg detection is more suitable for immunocompromised, hemodialysis, organ transplant patients. HCV‐cAg also can be used to monitor antiviral efficacy and predict sustained virological response (SVR).

### 2.2 HCV‐cAg can shorten the window period comparing with anti‐HCV antibody

**FIGURE 1:**
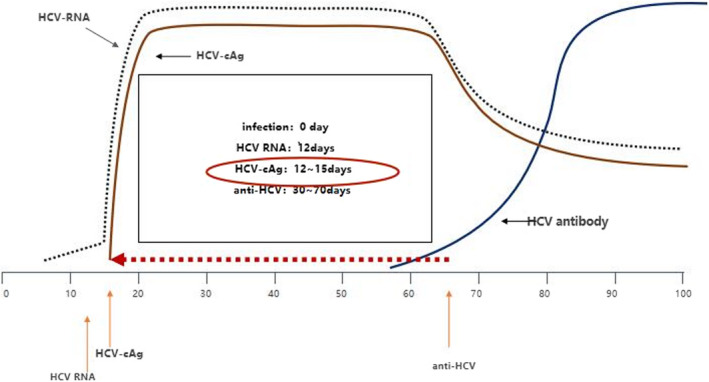
Emergence of laboratory markers for HCV infection. This image describes the time point at which various markers of infection (HCV‐RNA, HCV‐CAG, and anti‐HCV) first appeared in the body of an HCV infection

Anti‐HCV antibody detection assays detect HCV infection after approximately 70 days, and it may be negative in the early stages of acute hepatitis.^8^ HCV‐cAg can be detected (within 2–3 weeks of infection) much earlier than anti‐HCV antibodies (the window period can be extended to 12 weeks), almost simultaneously with HCV‐RNA^9^ (Figure 1). The positive results of HCV‐cAg confirmed the replication activity of the virus, can shorten the window period of HCV infection by 5~7 weeks.

A rapid diagnostic assay (*HCV Ag*‐*Ab Combo*) for simultaneous detection of HCV‐cAg and anti‐HCV antibodies for early diagnosis of HCV infection was developed.^10^ It detected HCV infection approximately 7–12 days earlier than the HCV‐Ab detection assays, and its performance was not affected when testing different genotypes of HCV. It could be a useful screening assay and an alternative to HCV‐RNA detection or HCV Ag‐Ab ELISA when nucleic acid technologies cannot be implemented.

### 2.3 HCV‐cAg expression highly agreement with that of HCV‐RNA

Following positive serology, the gold standard confirmatory test of HCV infection is detection of HCV‐RNA by PCR. However, HCV qRT‐PCR is expensive, time‐consuming, and requires advanced technical skills and equipment. A cheaper and quicker assay for initial testing for suspected HCV is needed, particularly in low‐resource settings.^11^ Less expensive and time‐consuming tests against HCV‐cAg have recently become available, which may be useful in diagnosing acute infection. The HCV‐cAg assay (ARCHITECT HCV Ag; Abbott Diagnostics) displays high sensitivity and strong correlation with HCV‐RNA (Abbott Real‐Time HCV assay; Abbott Molecular). HCV‐cAg can be used as a marker of viremia, as there is good nonlinear correlation with HCV‐RNA (*r* = 0.87 *vs* Abbot Real‐Time qRT‐PCR) with the lower limit of detection corresponding to the HCV‐RNA load of 700–1100 IU/mL.^12^ The ARCHITECT HCV Ag Assay is cheaper, faster (40 min), and simple to perform and has good sensitivity in samples with viral loads above 10,000 IU/mL.^6^

Anti‐HCV antibody (using ARCHITECT anti‐HCV assay; Abbott Diagnostics), HCV‐cAg (using ARCHITECT HCV Ag), and HCV‐RNA (using Abbott Real‐Time HCV assay) were detected in 298 seropositive subjects. Among them, 252 were positive for anti‐HCV antibody (signal‐to‐cutoff ratios ≥5), 220 were positive for HCV‐cAg, and 222 were detected with HCV‐RNA. HCV‐cAg significantly correlated with HCV‐RNA. The accuracy, sensitivity, specificity, positive predictive value, and negative predictive value of HCV‐cAg (≥3 fmol/L) to predict HCV viremia were 99%, 99%, 100%, 100%, and 97%, respectively.^13^ These results concluded that HCV‐cAg was a good surrogate marker for HCV‐RNA and could be used to diagnose active HCV infection in a resource‐limited setting. And using HCV‐cAg test to screen and identify active HCV carriers, which can reduce the cost of detection, would enable more patient access to efficacious and increasingly affordable direct‐acting antivirals (DAAs) for the treatment of HCV infection.

A two‐phase community‐based hepatitis C screen in a HCV‐prone area of central Taiwan was performed. The positive rate of anti‐HCV (using Roche Diagnostics GmbH) and HCV‐cAg (using ARCHITECT HCV Ag) for 935 training phase participants were 18.7% (175/935) and 8.3% (78/935), respectively. Using HCV‐RNA (COBAS AmpliPrep/COBAS TaqMan HCV Quantitative Test, version 2.0) as a gold standard, the sensitivity, specificity, and accuracy of HCV‐cAg were 97.1%, 98.6%, and 97.8%, respectively. During the validation phase, the positive predictive value (PPV) and negative predictive value (NPV) were 98.4% and 99.3%, respectively. Across the entire participant sample, a significant linear correlation between HCV‐cAg and HCV‐RNA concentration was noted (*r* ^2^ = 0.93, *p* < 0.001) following log‐log transformation.^14^

HCV‐RNA (Shanghai ZJ Bio‐Tec Co., Ltd) and HCV‐cAg (ARCHITECT HCV Ag) were detected in 229 serum samples which were positive for anti‐HCV antibody test. With HCV‐RNA assays as gold standard, the diagnostic sensitivity, specificity, positive and negative predictive values, and accuracy rate of HCV‐cAg assay were detected as 94.82%, 100%, 100%, 60%, and 95.2%, respectively. The area under the receiver operator characteristic (ROC) curve of HCV‐cAg was 0.989. 1 pg/mL of total HCV‐cAg is found to be equivalent to approximately 6607 HCV‐RNA IU/mL, which implied the expression level of HCV‐cAg was highly consistent with that of HCV‐RNA.^15^ Viral load (VL) tended to be lowest when anti‐HCV and HCV‐cAg were absent and to be highest when the antigen was detectable. Then, it decreased when anti‐HCV appeared at a level detectable by sensitive third generation tests.^16^

Using of HCV‐cAg alone for patients with a very low VL (VL <3000 IU/mL) may has a false‐negative result, which have enraised concerns.^17^ But, the benefits of the HCV‐cAg assay as a rapid, point‐of‐care test in population‐based screening still outweigh the risk of a small number of false negatives in a subset of patients since the prevalence of very low VL cohorts is <5% for now.^18^

Currently, RNA‐PCR tests are the gold standard. However, there is potential for the use of simpler and cheaper HCV‐cAg tests to confirm HCV infection in different clinical settings.^19^ The percentage HCV‐RNA negative in HCV‐cAg‐positive results was 0.57%, while the percentage HCV‐RNA positive with HCV‐antigen negative was 3.52% in 24 datasets including 8136 samples.^19^ Since there is strong evidence that antigen detection performs as well as RNA‐based assays for HCV management, the use of HCV‐cAg testing saves testing costs and increases the access of infected persons to HCV treatment.

Dried blood spot (DBS) cards of 68 patients with chronic HCV infection were collected and stored at different temperatures (−80, 4, 21, 37°C) for one week, and alternating 37, and 4°C, to assess whether temperature change during transportation would affect sensitivity. Sensitivity of HCV‐cAg assay (ARCHITECT HCV Ag) was as follows: 94% (−80°C), 94% (4°C),91% (21°C), 93% (37°C), and 93% (37°C/4°C),^20^ which concluded that temperature did not greatly affect the sensitivity of HCV‐cAg assay and supported the use of this assay as an alternative to HCV‐RNA.

The sensitivity and specificity of HCV‐cAg (ARCHITECT HCV Ag) were 76.6% and 100%, respectively, compared with HCV‐RNA (Roche Diagnostics) in 204 serum samples, and the correlation between the two techniques was extremely well (Pearson coefficient = 0.951). Although the HCV‐cAg assay was less sensitive than the HCV‐RNA test (HCV‐cAg was unable to detect viral loads below 5000 IU/mL), the correlation between both assays was excellent. Therefore, in emergency situations (such as screening of organ donors, low‐income settings, or small and low‐skilled laboratories with a limited workload et al), HCV‐cAg may be useful as an alternative diagnostic method for RNA detection in the diagnosis of acute or chronic HCV infection.^21^ Sensitivity of HCV‐cAg detection cannot be compared with that of RNA, which is especially important in samples with a low viral load. However, this drawback becomes less relevant and the correlation between both techniques is high when the VL over a specific value. Although the sensitivity for detecting low HCV‐RNA VL was not optimal, the HCV‐cAg showed excellent specificity and PPV.^21^

The sensitivity and specificity of HCV‐cAg (ARCHITECT HCV Ag) were 93.4% and 98.8% from bivariate analyses, while in quantitative studies using, HCV‐cAg was highly agreement with HCV‐RNA levels >3000 IU/mL. HCV‐cAg assays for signal amplification have the potential to replace nucleic acid testing in settings with high HCV prevalence since it has high sensitivity, high specificity, and good correlation with HCV‐RNA levels >3000 IU/mL.^17^

Gray zone results (GzR) were detected in 33 samples when using the HCV‐cAg assay (ARCHITECT HCV Ag) to assessment HCV‐cAg levels in 952 serum samples. In GzR, 25 were reactive on retesting and among which 19 were anti‐HCV positive (17 of these 19 samples were tested for HCV‐RNA [Roche COBAS AmpliPrep COBAS TaqMan HCV Quantitative Test. ver. 2.0] and were all reactive with viral loads <104 IU/mL). The remaining 6 samples were both anti‐HCV non‐reactive and HCV‐RNA undetectable. The other 8 GzR samples were non‐reactive on retesting and on HCV‐RNA test.^22^ No significant differences were found on comparing HCV‐cAg values. This confirms that, even though GzR occur, HCV‐cAg is a robust alternative to HCV‐RNA detection in the active detection of infections, except in donor screening scenarios, where the use of molecular methods would be advisable.

To evaluate the diagnostic efficacy of HCV‐cAg detection for HCV infection, 221 anti‐HCV‐positive serums was follow‐up tested by both HCV‐cAg (ARCHITECT HCV Ag) and HCV‐RNA (Cobas AmpliPrep/Cobas TaqMan HCV kit). Comparing with HCV‐RNA, the sensitivity and specificity for HCV‐cAg in predicting infection were 99% and 100%, respectively. A strong correlation between the assay of HCV‐RNA and HCV‐cAg (*r* = 0.960, *p* < 0.001) was revealed by this analysis of 221 anti‐HCV‐positive patient sera, which proposed that HCV‐cAg immunoassay is a more cost and time efficient alternative to the current diagnostic process.^23^

The diagnostic value of HCV‐cAg (ARCHITECT HCV Ag) as a detection tool was assessed using HCV NAT as the gold standard in serum samples, which were tested at a single laboratory in Scotland from June 2011 to December 2017. Among detection of 744 patients, the sensitivity and specificity of HCV‐cAg were 82.1% and 99.8%, respectively. Genotype 3 has found to be associated with increased odds of a false‐negative result (odds ratio(OR) = 3.59, 95% CI: 1.32–9.71), while older age (OR = 0.92, 95% CI: 0.88–0.97 per year) and VL (OR = 0.10, 95% CI: 0.05–0.21 perlog_10 _IU/mL) were associated with reduced odds of a false negative.^24^ Although HCV‐cAg testing apply to diagnosis could lead to significant cost savings in national screening programmes, a significant proportion of HCV‐infected individuals may be missed particularly in low‐ and middle‐income regions, where genotype 3 is prevalent. These findings have implications for HCV diagnosis and determination of viral clearance after treatment.

Replacing HCV‐RNA with HCV‐cAg for confirmation of positive anti‐HCV antibodies identified fewer active infections (−110 per 100,000 screened subjects) at significantly reduced total costs (‐$2.74 per screened) and costs per diagnosed infection (‐$44), which proved that HCV‐cAg assay can diagnosis chronic HCV‐infected patients effectively at low costs. Compared to the standard (Ab followed by RNA), adding a subsequent RNA confirmatory test on HCV‐cAg undetected samples captured at least the same rate as still reduced costs (–$1.16 per subject screened, −$22 per case detected).^25^ A combined pattern of anti‐HCV antibodies in screening followed by sequential confirmation with HCV‐cAg and HCV‐RNA in HCV‐cAg negatives would provide equal or better diagnostic performance at lower cost over abroad range of scenarios.

The positive results of HCV‐cAg test (Laibo Biology Science and Technology Co., Ltd.) have a high coincidence rate with HCV‐RNA, and can reflect the degree of liver function injury. HCV‐cAg test has an important value in auxiliary clinical diagnosis and treatment.^26^ The positive and negative coincidence rates of HCV‐cAg test (Jingda Biological Engineering Co., Ltd) and HCV‐RNA were 94.00% (47/50) and 96.67% (58/60), respectively, and the total coincidence rate was 95.45% (105/110).^27^ The sensitivity and accuracy of HCV‐cAg test (Keshun Biological Technology Co., Ltd) were higher than those of HCV‐Ab (*p* < 0.05).^28^ The results of HCV‐cAg test (Laibo Biology Science and Technology Co., Ltd) and HCV nucleic acid test were compared and analyzed. There was no significant difference between the two groups (*p* > 0.05). HCV core antigen detection was significantly better than anti‐HCV detection (*p* < 0.05). Based on the analysis of various factors, it is concluded that the detection of HCV core antigen is economical and practical, and the operation is simple, and can be used as an effective means to screen HCV infection.^29^

There are different platforms of conducting HCV core antigen testing; among all, Abbott ARCHITECT HCV Ag assay has the best quality and used by most studies. The HCV core antigen testing, using different platforms, showed consistent results of high sensitivity and specificity as compared to gold standard HCV‐RNA. Diagnostic value of HCV‐cAg detection comparing with HCV‐RNA is showed in Table 1.

**TABLE 1:** Diagnostic value of HCV‐cAg detection (comparing with HCV‐RNA)

Researchers	Sensitivity	Specificity	Positive Predictive Value	Negative Predictive Value	Sample size (*n*)	Qualitative or quantitative
Rujipat Wasitthankasem^13^	99%	100%	100%	97%	270	Quantitative
Lijuan Wang^15^	94.82%	100%	100%	60%	125	Quantitative
R. Alonso^30^	96.2%	100%	100%	96.2%	103	Quantitative
Wei‐Ming Chen^14^	97.1%	98.6%	98.4%	99.3%	1220	Qualitative
Fiona V. Cresswell^31^	100%	97.96%	88%	100%	15	Quantitative
Hullegie SJ^32^	89%	100%	100%	82.18%	57	Qualitative
Kevin G. Pollock^24^	82.1%	99.8%	99.58%	89.7%	744	Qualitative
Emily Adland^33^	94.5%	100%	100%	90.5%	305	Quantitative
Duchesne L^34^	95.7%	99.7%	98.1%	99.6%	1037	Quantitative
Xue Zheng Wong^35^	90.7%	100%	100%	76.5%	112	Qualitative
Alonso R^21^	76.6%	100%	100%	41.4%	204	Quantitative
Christine Chang^23^	99.0%	100%	100%	91.7%	221	Quantitative

### 2.4 HCV‐cAg detection has advantages for people with immunodeficiency or at high risk

Immunosuppressed or immunosuppressed patients may be tested negative for anti‐HCV antibodies.^8^ HCV is increasingly common among human immunodeficiency virus (HIV)‐infected men who have sex with men.^36^ Affordable and sensitive screening methods for acute HCV are necessary to successfully intervene in the current HCV epidemic among HIV‐positive men having sex with men. One study showed that HCV‐cAg proved sensitive (100%) and specific (97.9%) in diagnosing acute HCV in a HIV‐infected cohort when compared with HCV PCR.^31^ In addition, routine use of HCV‐cAg detection in screening tests instead of QRT‐PCR can save potential costs. The cost per individual screen would be approximately $85 less ($108 for qRT‐PCR *vs* $23.4 for HCV core antigen including kit, staff, and laboratory extras, although this will vary between laboratories).^31^ As a quick, simple, and cost‐effective test, HCV‐cAg has considerable utility in screening for acute HCV.

Another research has evaluated the diagnostic effectiveness of HCV‐cAg testing in acute HCV‐infected HIV‐positive patients. HCV‐cAg was identified in 39 out of 44 patients with detectable HCV‐RNA levels and undetectable in all 23 control patients without detectable HCV‐RNA in plasma, which resulted in a sensitivity and specificity of HCV‐cAg of respectively 89% and 100%. Furthermore, the correlation between HCV‐cAg and HCV‐RNA was 0.97 (*p* < 0.001) upon diagnosis.^32^ The data presented in this study suggest that HCV‐cAg testing is a sensitive and specific method that can be used in diagnosing acute HCV in HIV‐infected patients.

HCV‐cAg, which had a sensitivity of 95.7% and a specificity of 99.7% in diagnosing chronic hepatitis C, corresponding to area under ROC (AUROC) of 0.99, was highly correlated with HCV‐RNA (the Spearman *ρ* correlation coefficient = 0.75 [*p* < 0.00001]). Being HIV‐ or HBV‐infected did not impact the performance of HCV‐cAg (sensitivity = 96.4%, specificity = 96.2% and AUROC = 0.98 in the HBV group, sensitivity = 100%, specificity = 88.2% and AUROC = 0.99 in the HIV group, *p* value between AUROC = 0.69).^34^ A high specificity and sensitivity for the diagnosis of chronic hepatitis C were displayed in HCV‐cAg quantification in Cameroon, and its performance was not significantly modified by a concomitant HIV or HBV infection. Using HCV‐cAg quantification as a screening tool to directly identify chronic hepatitis C could be a reliable tool in a "test and treat" strategy in the context of chronic hepatitis C elimination on a global scale.

Diagnose a HCV infection is important to reduce the incidence of this infection in hemodialysis (HD) patients, which group is at a higher risk of contracting hepatitis C. A cross‐sectional study was conducted to assess the correlation between HCV‐cAg and HCV‐RNA among HD patients. HCV‐cAg correlates well with HCV‐RNA (Spearman test coefficient 0.833, *p* < 0.001), with sensitivity of 90.7%, specificity 100%, PPV 100%, negative predictive value (NPV) 76.5%, and accuracy 92.9%. With the increase of HCV‐RNA level, the sensitivity and correlation were better (HCV‐RNA level >3000 IU/mL, HCV‐cAg sensitivity was 95.1% and Spearman test coefficient 0.897 [*p* < 0.001]).^35^ Test of 98 samples from HCV‐infected patients pointed out that the HCV Ag and HCV‐RNA results agreed well (Spearman test coefficient 0.9041, *p* < 0.0001) in all genotypes and subtypes for HD patients.^37^ Another study found that the sensitivity, specificity, and accuracy of the combined Ag/Ab test applied for early detection of HCV infection among HD patients were higher than that of anti‐HCV antibodies detection test (95.45%, 94.1%, and 94.87% vs 81.8%, 88.23%, and 84.6%), which means that the combined Ag/Ab test can be as an alternative to HCV‐RNA detection.^38^ Since HCV‐cAg shows an excellent correlation with HCV‐RNA and has 100% PPV, it can be considered as an alternative diagnostic tool for chronic active HCV infection among HD cohort. HCV‐RNA test only commends to follow up when seropositive HD patient with HCV‐cAg undetected.

The survival rate of the HCV‐cAg‐positive recipients decreased rapidly at 240 months after the renal transplant procedure, which was clarified by a cohort study on the long‐term outcomes of Japanese renal allograft recipients with HCV infections. In addition, a Cox proportional hazards model indicated that positivity for the HCV‐cAg was the most important independent risk factor for post‐renal transplant survival and allograft function [survival: hazard ratio (HR) 3.93 (1.54–10.03), *p* = 0.004; graft function: HR 2.09 (1.14–3.81), *p* = 0.016].^39^

In summary, there is a higher risk of HCV infection in some populations, such as injecting drug users (IDUs),^40^ hemodialysis patients (due to the procedure rather than the dialysis itself) and in HIV‐positive individuals et al.^36^ Further evaluation of the high‐risk populations confirmed that the determination of the HCV‐cAg could significantly predict the diagnosis of acute hepatitis C with an accuracy similar to that of HCV‐RNA.^31^

### 2.5 HCV core antigen can distinguish between previous HCV infection or current infection

Detection of HCV active infection without clinical manifestations is the first and crucial step to prevent further spread of infection and improve the health status of those already infected.^41^ One limitation of anti‐HCV antibody testing is that a positive result does not distinguish the stage of HCV infection (inactive, active, acute, or chronic), because it only shows previous infection with HCV and does not provide additional information indicating the current infection status. Anti‐HCV antibodies can still be detected in subjects who have cleared the infection and no longer carry the virus, as the elimination of infection can not only be achieved through effective treatment, but also can occur spontaneously.^42^ Evidence from a recent population study conducted in European hospitals suggests that the frequency of active HCV infection in asymptomatic patients with positive anti‐HCV is <50%.^43^ HCV‐cAg is a serological indicator of viral replication, which can distinguish previous infection of HCV or current infection, and is highly consistent with the detection results of HCV‐RNA. Therefore, the equivalence of HCV‐RNA and HCV‐cAg for diagnostic purposes has been approved by the European Association for the Study of the Liver (EASL).^8^

This pilot study demonstrated the potential for HCV‐cAg testing as a reflex test to discriminate between active or past infection for seropositive individuals.^44^ HCV‐cAg testing has been suggested to provide a cheaper and more rapid turnaround time to issue results compared to RNA testing.^31^ Use of HCV‐cAg testing may be an important component for HCV screening enabling the potential for earlier diagnosis, linkage to care and commencement of treatment.^31^, ^45^

The anti‐HCV antibody test had a higher false positive, with a positive predictive value of 87% compared to the repeated HCV‐Ab testing in the reference laboratory.^33^ However, HCV‐cAg was proved to have 100% positive predictive value compared to detection of HCV‐RNA. There was a strong correlation between quantitative HCV‐cAg and HCV‐RNA viral load (*p* < 0.0001), which make HCV‐cAg perform well as a diagnostic test compared to HCV‐RNA.^33^ This result indicates that in the case of difficulty in carrying out HCV‐RNA detection, HCV‐cAg may be a good alternative test, particularly at higher viral loads (e.g., if HCV‐RNA >104 IU/mL).

To assess the feasibility of HCV‐cAg assay in community screening programmes, a study has included 2027 volunteers. The agreement between HCV‐cAg and HCV‐RNA was 100% in anti‐HCV‐positive group, and the correlation of HCV‐cAg with HCV‐RNA (*R* ^2^ = 0.84, *p* < 0.005) was good.^46^ The utility of HCV‐cAg testing to confirm active infection in screening programs was highlighted by the agreement between HCV‐RNA and HCV‐cAg.

### 2.6 Prediction of Treatment Response in Patients with Chronic Hepatitis C

The high effectiveness of DAAs and its extreme safety issues requires monitoring throughout the treatment period. To evaluate the diagnostic usefulness of a HCV‐cAg assay in HCV‐infected patients undergoing treatment with DAAs, researchers analyzed 103 samples from 28 patients. Sensitivity and specificity were 96.2% and 100% of HCV‐cAg assay comparing with RT‐PCR, which shows that HCV‐cAg is a reliable marker for the follow‐up of HCV‐infected patients undergoing treatment with DAAs.^30^ The decreasing and increasing curves for RT‐PCR and HCV Ag during treatment were almost the same. Therefore, both assays were excellent predictors of the success or failure of treatment.

It has been proposed that HCV‐cAg assay was a more economical alternative compared to HCV‐RNA detection. A study devoted to investigate the clinical utility of HCV‐cAg assay in the monitoring of DAAs for chronic hepatitis C patients found that baseline HCV‐cAg levels showed good correlations with HCV VL (*r* = 0.879; *p* < 0.001). The consistent rate between HCV‐cAg negative with HCV‐RNA undetectable was significantly better in the COBAS TaqMan HCV (CTM) test than in the Abbott Real‐Time (ART) HCV assay at week 2 (*p* = 0.003) and week 4 (*p* = 0.003). The HCV‐cAg assay identified 99% of who has achieved aSVR 12 weeks off therapy (SVR12) among 108 patients. Both HCV‐cAg and HCV‐RNA undetectability in serum had high PPV at week 2 (98% vs 100%) and at week 4 (97% vs 99%) in predicting SVR12, which supposed that HCV‐cAg assay may be a feasible alternative to HCV NAT for the determination of SVR12 in patients treated with DAAs.^47^

Levels of HCV‐cAg were determined at baseline in 92 patients with HCV infection who had been treated with pegylated interferon and ribavirin and at week 4 in 15 patients. There is a good correlation between baseline HCV‐cAg levels and HCV‐RNA (*r* = 0.79, *p* < 0.001). Based on HCV‐RNA analysis, mean HCV‐cAg levels at baseline were significantly lower in patients with a SVR than in those with a non‐SVR (non‐responder or relapse) (2.8 log_10_ fmol/L vs 3.27 log_10_ fmol/L, *p* = 0.023). HCV‐RNA and HCV‐cAg levels have similarly shaped curves in monitoring of the viral kinetics under treatment. Based on HCV‐RNA assay results, patients with undetectable HCV‐cAg levels at week 4 had a 92.3% probability of achieving SVR.^48^ These results conducted that HCV‐cAg assay may be used as a supplement for predicting treatment response in HCV infection, but not as an alternative to the HCV‐RNA assay.

HCV‐cAg and HCV‐RNA were tested in plasma or serum samples from three patient groups: new diagnosis, monitoring treatment, and therapeutic failure in a prospective study which was carried out in a regional referenced hospital in Spain. The beginning of the treatment, four weeks post‐initiation, at the end, and 12 weeks posttreatment finalization were tested in monitoring treatment group. A total of 303 samples from 124 patients were analyzed. HCV‐cAg sensitivity and specificity were 97% and 95%, respectively, while the optimal cutoff value was 3 fmol/L in the ROC analysis and the area under the curve was 0.987 (0.972–1.000). HCV‐cAg was excellent correlated with HCV‐RNA (*R* ^2^ = 0.932).^49^ The highly agreement between HCV‐cAg and HCV‐RNA may allow, reducing follow‐up costs in patients diagnosed with HCV and of the DAA treatment, as well as faster and easier results. As a marker for active HCV infection, new diagnosis, detection of antiviral therapeutic failure, and treatment monitoring, HCV‐cAg demonstrated good sensitivity and specificity.

To evaluate the clinical performance of HCV‐cAg assay from plasma samples to monitor HCV treatment efficacy and HCV viral recurrence, HCV‐cAg and HCV‐RNA were detected at baseline, end of treatment response, and SVR visits. The sensitivity of the HCV‐cAg assay with quantifiable HCV‐RNA threshold was 94% (95% CI: 88%, 98%), 56% (21%, 86%), and 100%, respectively, while the specificity was between 98% and 100% for all time points assessed. All six participants with viral recurrence have been detected accurately by HCV‐cAg, demonstrating 100% sensitivity and specificity. One participant with detectable (non‐quantifiable) HCV‐RNA and non‐reactive HCV‐cAg at SVR12 subsequently cleared HCV‐RNA at SVR24.^50^ This study indicates that confirmation of active HCV infection, including recurrent viraemia, by HCV‐cAg is possible, since HCV‐cAg demonstrated high sensitivity and specificity for detection of pre‐treatment and posttreatment viraemia. Reduced on‐treatment sensitivity of HCV‐cAg may be a clinical advantage given the moves toward simplification of monitoring schedules.

It is estimated that 90% (RNA >10,000 IU/mL) of the positive HCV‐RNA samples falls in the sensitivity range of HCV‐cAg assays, which make the HCV‐cAg test to be a cost‐effective method for confirming HCV infection compared to HCV‐RNA and also been proposed as a substitute for HCV‐RNA in determining SVR.^51^

HCV‐RNA and HCV‐cAg have high predictive value for sustained virological response (SVR).^52^ In addition, HCV‐cAg expression was highly consistent with RNA quantitative test results, and the measurement of HCV‐cAg in the recent EASL guidelines is a potential alternative method for monitoring treatment response in DAA‐based treatment regiments.^8^

### 2.7 Use of hepatitis C core antigen qualitative and quantitative tests

The qualitative test of HCV core antigen can be used for but not limited to the diagnosis of acute hepatitis C virus infection in the window period, the differential diagnosis of past infection and present infection, and the diagnosis of acute hepatitis C virus infection in immunosuppressed population and high‐risk population. Since the expression of HCV core antigen is highly consistent with the viral load of HCV‐RNA, HCV core antigen quantitative assay can be used as a substitute for HCV‐RNA for baseline viral load analysis before antiviral therapy and response assessment during and after antiviral therapy.

### 2.8 Cost‐effectiveness of HCV‐cAg test

Data showed that the introduction of HCV‐cAg test, if compared with the standard one, would give similar effectiveness, with a lower organizational and economic impact (Economic costs for the hospital and Regional and National Health Service would be saved: A hospital could reduce the direct and indirect costs by 47.90% each and contribute to a reduction in funding at Regional or National level by 26.96%), with a good equity impact for HCV‐infected patients.^53^ In addition, the time needed to complete a medical report by HCV‐cAg test was reduced to 90 minutes, and allowed an immediate definition of the presence or absence of HCV infection, which has a positive organizational impact.^53^ Kadkhoda K pointed out that HCV core antigen used to diagnose HCV infection has a significant cost‐ (a minimum of 52.13% reduction in costs compared to qualitative RNA testing), labor‐, and turnaround time‐reducing potential.^54^ Although compared with HCV‐RNA, the missed detection rate of HCV‐cAg was 0.11% (110/100,000 screened subjects), but total costs reduced $2.74 per screened and costs per diagnosed infection reduced $44. Adding subsequent HCV‐RNA validation test to the HCV‐cAg‐negative sample to achieve at least the same rate of RNA after anti‐HCV still reduced $1.16 per screening subject and $22 per test case.^25^ WHO also signed that instead of HCV‐RNA with HCV‐cAg testing in acute HCV infection would reduce a few thousand dollars cost (attains the same sensitivity), and saved time effectively. Another significant advantage of HCV Ag is that it can be measured in the same laboratory department using the same analytical system as anti‐HCV. And there is no need to increase the cost of additional equipment and laboratory setup.^43^ Therefore, a combination of antibody and antigen tests for screening, followed by RNA confirmation of antibody‐positive Ag‐negative samples, can provide equal or better diagnostic performance in a variety of situations at a lower cost.

## 3 CONCLUSION

On the one hand, HCV‐cAg appears earlier than anti‐HCV antibodies, is expressed only in active HCV infection, can differentiate between previous infection and current infection, and is not interfered by immunosuppressed or immunosuppressed patients. On the other hand, the expression of HCV‐cAg is highly consistent with that of HCV‐RNA, but compared with HCV‐RNA, detection of HCV‐cAg is easy to operate, time saving, and low cost. Therefore, HCV core antigen has similar clinical sensitivity to NAT and can be used as a substitute for HCV‐RNA in the diagnosis of virus infection.^3^ Combined detection of HCV‐cAg and antibody serology can help doctors detect HCV infection earlier, accurately diagnose different stages of HCV infection, and evaluate the therapeutic effect of antiviral drugs, which are beneficial in the prevention and treatment of hepatitis C.

## CONFLICTS OF INTEREST

There is no conflicts interest to report.

## AUTHOR CONTRIBUTIONS

Wang Yuhan participated in analysis and interpretation of data. Wang Yuhan, Wang Jie, and Jiang Ling drafted the article. Huang Yuanshuai revised it for critically important intellectual content. Wang Yuhan, Wang Jie, Jiang Ling, and Huang Yuanshuai participated in the conception and design of the article and finally approved the version to be published.

## Data Availability

All data generated or used during the study appear in the submitted article.

## References

[1] Global prevalence and genotype distribution of hepatitis C virus infection in 2015: a modelling study. Lancet Gastroenterol Hepatol. 2017;2:161‐176. 28404132 10.1016/S2468-1253(16)30181-9

[2] Rafik M , Bakr S , Soliman D , et al. Characterization of differential antibody production against hepatitis C virus in different HCV infection status. Virol J. 2016;13:116. 27357382 10.1186/s12985-016-0572-9 PMC4928299

[3] Guidelines for the care and treatment of persons diagnosed with chronic hepatitis C virus infection. Geneva: © World Health Organization; 2018. 30307724

[4] Galli C , Julicher P , Plebani M . HCV core antigen comes of age: a new opportunity for the diagnosis of hepatitis C virus infection. Clin Chem Lab Med. 2018;56:880‐888. 29702484 10.1515/cclm-2017-0754

[5] Contreras AM , Tornero‐Romo CM , Toribio JG , et al. Very low hepatitis C antibody levels predict false‐positive results and avoid supplemental testing. Transfusion. 2008;48:2540‐2548. 18680546 10.1111/j.1537-2995.2008.01886.x

[6] Benito R , Arribas J , Algarate S , Cebollada R , Gude MJ . Hepatitis C virus core antigen for screening organ donors and recipients. Diagn Microbiol Infect Dis. 2018;91:126‐129. 29477273 10.1016/j.diagmicrobio.2018.01.021

[7] Schüttler CG , Thomas C , Discher T , et al. Variable ratio of hepatitis C virus RNA to viral core antigen in patient sera. J Clin Microbiol. 2004;42:1977‐1981. 15131157 10.1128/JCM.42.5.1977-1981.2004 PMC404599

[8] EASL recommendations on treatment of hepatitis C 2018. J Hepatol. 2018;69:461‐511. 29650333 10.1016/j.jhep.2018.03.026

[9] Laperche S , Nübling CM , Stramer SL , et al. Sensitivity of hepatitis C virus core antigen and antibody combination assays in a global panel of window period samples. Transfusion. 2015;55:2489‐2498. 26013970 10.1111/trf.13179 PMC4744653

[10] Patel J , Sharma P . Design of a novel rapid immunoassay for simultaneous detection of hepatitis C virus core antigen and antibodies. Arch Virol. 2020;165:627‐641. 31965313 10.1007/s00705-019-04518-0

[11] Chakravarti A , Chauhan MS , Dogra G , Banerjee S . Hepatitis C virus core antigen assay: can we think beyond convention in resource limited settings? Braz J Infect Dis. 2013;17:369‐374. 23602467 10.1016/j.bjid.2012.10.028 PMC9427406

[12] Medici MC , Furlini G , Rodella A , et al. Hepatitis C virus core antigen: analytical performances, correlation with viremia and potential applications of a quantitative, automated immunoassay. J Clin Virol. 2011;51:264‐269. 21621454 10.1016/j.jcv.2011.05.003

[13] Wasitthankasem R , Vichaiwattana P , Auphimai C , et al. HCV core antigen is an alternative marker to HCV RNA for evaluating active HCV infection: implications for improved diagnostic option in an era of affordable DAAs. PeerJ. 2017;5:e4008. 29134150 10.7717/peerj.4008 PMC5678506

[14] Chen WM , Lee CY , Hsu NT , et al. Feasibility of anti‐HCV reflex HCV Ag screening strategy in an HCV endemic community. J Formos Med Assoc. 2020. 10.1016/j.jfma.2020.09.013 33008696

[15] Wang L , Lv H , Zhang G . Hepatitis C virus core antigen assay: an alternative method for hepatitis C diagnosis. Ann Clin Biochem. 2017;54:279‐285. 27614354 10.1177/0004563216661218

[16] Grabarczyk P , Kubicka‐Russel D , Kopacz A , et al. Seronegative hepatitis C virus infection in Polish blood donors‐Virological characteristics of index donations and follow‐up observations. J Med Virol. 2020;92:339‐347. 31670401 10.1002/jmv.25617 PMC7003774

[17] Freiman JM , Tran TM , Schumacher SG , et al. Hepatitis C core antigen testing for diagnosis of hepatitis C virus infection: a systematic review and meta‐analysis. Ann Intern Med. 2016;165:345‐355. 27322622 10.7326/M16-0065 PMC5345254

[18] Mathur P , Kottilil S . Hepatitis C core antigen testing: still an effective diagnostic method for global elimination of hepatitis C. Clin Infect Dis. 2020;70:674‐675. 30943285 10.1093/cid/ciz273 PMC8204483

[19] Khan H , Hill A , Main J , Brown A , Cooke G . Can hepatitis C virus antigen testing replace ribonucleic acid polymearse chain reaction analysis for detecting hepatitis C virus? A systematic review. Open Forum Infect Dis. 2017;4:ofw252. 28567430 10.1093/ofid/ofw252 PMC5445222

[20] Biondi MJ , van Tilborg M , Smookler D , et al. Hepatitis C core‐antigen testing from dried blood spots. Viruses. 2019;11(9):830. 10.3390/v11090830 PMC6784259 31489933

[21] Alonso R , Perez‐Garcia F , Lopez‐Roa P , Alcala L , Rodeno P , Bouza E . HCV core‐antigen assay as an alternative to HCV RNA quantification: a correlation study for the assessment of HCV viremia. Enferm Infecc Microbiol Clin. 2018;36:175‐178. 10.1016/j.eimc.2016.11.013 28245938

[22] Arribas J , Benito R , Cebollada R , et al. Implications of grey zone results for routine hepatitis C virus screening with the ARCHITECT HCV‐Ag assay. J Appl Microbiol. 2020;128:899‐906. 31713922 10.1111/jam.14517

[23] Chang C , Hung CH , Wang JH , Lu SN . Hepatitis C core antigen highly correlated to HCV RNA. Kaohsiung J Med Sci. 2018;34:684‐688. 30527202 10.1016/j.kjms.2018.08.002 PMC11915677

[24] Pollock KG , McDonald SA , Gunson R , et al. Real‐world utility of HCV core antigen as an alternative to HCV RNA testing: Implications for viral load and genotype. J Viral Hepat. 2020;27:996‐1002. 32479681 10.1111/jvh.13337

[25] Julicher P , Galli C . Identifying cost‐effective screening algorithms for active hepatitis C virus infections in a high prevalence setting. J Med Econ. 2018;21:1‐10. 28881157 10.1080/13696998.2017.1369983

[26] Liu Y , ZQaLL . The clinical value of HCV‐CAG test and its relationship with HCV‐RNA, ALT and AST. J Clin Transfus Lab Med. 2020;22:316‐318.

[27] Gu Guoning JX , Hongjia Z . Application of hepatitis C virus core antigen detection in the diagnosis of hepatitis C patients. Chin J Mod Drug Appl. 2018;12:39‐41.

[28] Dongdong L . Value of detection of hepatitis C antibody and core antigen in diagnosis of hepatitis C. Henan J Prev Med. 2020;31:318‐320.

[29] Weifang L . Application value of core antigen detection in the diagnosis of HIV/AIDS combined with hepatitis C infection. World's Lat Med informat Dig. 2016;16:121‐122.

[30] Alonso R , Pérez‐García F , Ampuero D , Reigadas E , Bouza E . New direct‐acting antivirals for patients with chronic HCV infection: can we monitor treatment using an HCV core antigen assay? Diagn Microbiol Infect Dis. 2017;87:243‐246. 27916546 10.1016/j.diagmicrobio.2016.11.010

[31] Cresswell FV , Fisher M , Hughes DJ , Shaw SG , Homer G , Hassan‐Ibrahim MO . Hepatitis C core antigen testing: a reliable, quick, and potentially cost‐effective alternative to hepatitis C polymerase chain reaction in diagnosing acute hepatitis C virus infection. Clin Infect Dis. 2015;60:263‐266. 25301216 10.1093/cid/ciu782

[32] Hullegie SJ , GeurtsvanKessel CH , van der Eijk AA , Ramakers C , Rijnders BJA . HCV antigen instead of RNA testing to diagnose acute HCV in patients treated in the Dutch Acute HCV in HIV Study. J Int AIDS Soc. 2017;20:21621. 28692208 10.7448/IAS.20.1.21621 PMC5515013

[33] Adland E , Jesuthasan G , Downs L , et al. Hepatitis virus (HCV) diagnosis and access to treatment in a UK cohort. BMC Infect Dis. 2018;18:461. 30217169 10.1186/s12879-018-3367-3 PMC6137907

[34] Duchesne L , Njouom R , Lissock F , et al. HCV Ag quantification as a one‐step procedure in diagnosing chronic hepatitis C infection in Cameroon: the ANRS 12336 study. J Int AIDS Soc. 2017;20:21446. 28530032 10.7448/IAS.20.1.21446 PMC5515056

[35] Wong XZ , Gan CC , Mohamed R , et al. Hepatitis C core antigen testing to diagnose active hepatitis C infection among haemodialysis patients. BMC Nephrol. 2020;21:480. 33187498 10.1186/s12882-020-02154-4 PMC7666439

[36] Vanhommerig JW , van de Laar TJ , Koot M , et al. Evaluation of a hepatitis C virus (HCV) antigen assay for routine HCV screening among men who have sex with men infected with HIV. J Virol Methods. 2015;213:147‐150. 25528203 10.1016/j.jviromet.2014.11.026

[37] Miedouge M , Saune K , Kamar N , Rieu M , Rostaing L , Izopet J . Analytical evaluation of HCV core antigen and interest for HCV screening in haemodialysis patients. J Clin Virol. 2010;48:18‐21. 20233674 10.1016/j.jcv.2010.02.012

[38] El‐Emshaty WM , Raafat D , Elghannam DM , Saudy N , Eltoraby EE , Metwalli AE . Diagnostic performance of an immunoassay for simultaneous detection of Hcv core antigen and antibodies among haemodialysis patients. Braz J Microbiol. 2011;42:303‐309. 24031636 10.1590/S1517-83822011000100039 PMC3768914

[39] Okino K , Okushi Y , Mukai K , et al. The long‐term outcomes of hepatitis C virus core antigen‐positive Japanese renal allograft recipients. Clin Exp Nephrol. 2017;21:1113‐1123. 28357506 10.1007/s10157-017-1394-9

[40] van de Laar TJ , Molenkamp R , van den Berg C , et al. Frequent HCV reinfection and superinfection in a cohort of injecting drug users in Amsterdam. J Hepatol. 2009;51:667‐674. 19646773 10.1016/j.jhep.2009.05.027

[41] Ward JW . Testing for HCV: the first step in preventing disease transmission and improving health outcomes for HCV‐infected individuals. Antivir Ther. 2012;17:1397‐1401. 23321543 10.3851/IMP2477

[42] Sarpel D , Baichoo E , Dieterich DT . Chronic hepatitis B and C infection in the United States: a review of current guidelines, disease burden and cost effectiveness of screening. Expert Rev Anti Infect Ther. 2016;14:511‐521. 27043049 10.1586/14787210.2016.1174066

[43] Bert F , Rindermann A , Abdelfattah MA , Stahmeyer JT , Rossol S . High prevalence of chronic hepatitis B and C virus infection in a population of a German metropolitan area: a prospective survey including 10 215 patients of an interdisciplinary emergency unit. Eur J Gastroenterol Hepatol. 2016;28:1246‐1252. 27439034 10.1097/MEG.0000000000000702

[44] Evans H , Balasegaram S , Douthwaite S , et al. An innovative approach to increase viral hepatitis diagnoses and linkage to care using opt‐out testing and an integrated care pathway in a London Emergency Department. PLoS One. 2018;13:e0198520. 30044779 10.1371/journal.pone.0198520 PMC6059401

[45] Tillmann HL . Hepatitis C virus core antigen testing: role in diagnosis, disease monitoring and treatment. World J Gastroenterol. 2014;20:6701‐6706. 24944462 10.3748/wjg.v20.i22.6701 PMC4051911

[46] Kyuregyan KK , Malinnikova EY , Soboleva NV , et al. Community screening for hepatitis C virus infection in a low‐prevalence population. BMC Public Health. 2019;19:1038. 31375104 10.1186/s12889-019-7388-7 PMC6679455

[47] Lin SF , Tung SY , Wei KL , et al. Clinical utility of hepatitis C virus core antigen assay in the monitoring of direct‐acting antivirals for chronic hepatitis C. PLoS One. 2020;15:e0229994. 32126125 10.1371/journal.pone.0229994 PMC7053745

[48] Kim MN , Kim HS , Kim JK , et al. Clinical utility of a new automated hepatitis C virus core antigen assay for prediction of treatment response in patients with chronic hepatitis C. J Korean Med Sci. 2016;31:1431‐1437. 27510387 10.3346/jkms.2016.31.9.1431 PMC4974185

[49] Perez‐Garcia A , Aguinaga A , Navascues A , Castilla J , Ezpeleta C . Hepatitis C core antigen: diagnosis and monitoring of patients infected with hepatitis C virus. Int J Infect Dis. 2019;89:131‐136. 31580940 10.1016/j.ijid.2019.09.022

[50] Lamoury FMJ , Soker A , Martinez D , et al. Hepatitis C virus core antigen: a simplified treatment monitoring tool, including for post‐treatment relapse. J Clin Virol. 2017;92:32‐38. 28521211 10.1016/j.jcv.2017.05.007

[51] Nguyen LT , Gray E , O'Leary A , Carr M , De Gascun CF . The role of hepatitis C virus core antigen testing in the era of direct acting antiviral therapies: what we can learn from the protease inhibitors. PLoS One. 2016;11:e0163900. 27711230 10.1371/journal.pone.0163900 PMC5053597

[52] Tedder RS , Tuke P , Wallis N , Wright M , Nicholson L , Grant PR . Therapy‐induced clearance of HCV core antigen from plasma predicts an end of treatment viral response. J Viral Hepat. 2013;20:65‐71. 10.1111/j.1365-2893.2012.01630.x 23231086

[53] Monari M , Foglia E , Montanelli A , et al. Economic, organizational and budget impact of a new diagnostic plan for HCV detection: what’s “new”? Rivista Italiana della Medicina di Laboratorio. 2015;11:236‐242.

[54] Kadkhoda K , Smart G . HCV antigen testing for the diagnosis of hepatitis C infection: a cost‐efficient algorithm. Clin Lab. 2014;60:677‐680. 24779304 10.7754/clin.lab.2013.130634

